# Perennial Flowering Plants Sustain Natural Enemy Populations in Gobi Desert Oases of Southern Xinjiang, China

**DOI:** 10.3390/insects13050399

**Published:** 2022-04-20

**Authors:** Yangtian Liu, Bing Liu, Qian Li, Mengxiao Sun, Minlong Li, Kris A. G. Wyckhuys, Peiling Wang, Yanhui Lu

**Affiliations:** 1 Key Laboratory of Oasis Agricultural Pest Management and Plant Protection Resources Utilization, College of Agriculture, Shihezi University, Shihezi 832003, China; yangtianlwmt@163.com; 2 State Key Laboratory for Biology of Plant Diseases and Insect Pests, Institute of Plant Protection, Chinese Academy of Agricultural Sciences, Beijing 100193, China; liubing1945@126.com (B.L.); liqian362771034@163.com (Q.L.); smx19921117@126.com (M.S.); li694449602@163.com (M.L.); k.wyckhuys@uq.edu.au (K.A.G.W.)

**Keywords:** biological control, insect biodiversity conservation, native plants, drought, desert ecosystems, bottom-up effects

## Abstract

**Simple Summary:**

Natural habitats are essential providers of biological conservation services. The crucial role of the Gobi Desert, a dominant landscape of desert-oasis ecosystems in natural predator conservation is poorly understood, especially in southern Xinjiang, China’s Tarim Basin, where the Gobi Desert is directly adjacent to farmland and characterized by extremely sparse vegetation and more severe climatic conditions. In this context, we investigated the floral composition of the Gobi Desert and gauged the identity, relative abundance, and temporal dynamics of predatory insects associated with the prevailing plant species. We also explored whether certain plant traits and herbivore abundance are related to either natural enemy identity or relative abundance. Our results demonstrate that perennial flowering plants, such as *Apocynum pictum* (Apocynaceae), *Phragmites communis* (Poaceae), *Karelinia caspia* (Asteraceae), and *Tamarix ramosissima* (Tamaricaceae), are the dominant species of vegetation community in the Gobi Desert, and could sustain diversified arthropod predators, i.e., ladybeetles, spiders, and other natural enemies. This work not only informs sustainable pest management initiatives, but also shows how non-crop habitats at the periphery of agricultural fields underpin ecological resilience under adverse climatic conditions.

**Abstract:**

Natural habitats play crucial roles in biodiversity conservation and shape the delivery of ecosystem services in farming landscapes. By providing diverse resources to foraging natural enemies, they can equally enhance biological pest control. In this study, we described the plant community and foliage-dwelling invertebrate predators within non-crop habitats of the Gobi Desert oases in southern Xinjiang, China. We assessed whether plant-related variables (i.e., species identity, flowering status) and herbivore abundance affect natural enemy identity and abundance. A total of 18 plant species belonging to 18 genera and 10 families were commonly encountered, with *Apocynum pictum* (Apocynaceae), *Phragmites communis* (Poaceae), *Karelinia caspia* (Asteraceae), and *Tamarix ramosissima* (Tamaricaceae) as the dominant species. Certain plant species (*P. communis*) primarily provide shelter, while others offer (floral, non-floral) food resources or alternative prey. Predatory ladybeetles and spiders were routinely associated with these plants and foraged extensively within adjacent field crops. Plant traits and herbivore abundance explained up to 44% (3%–44%) variation in natural enemy community and exhibited consistent, year-round effects. Among all plant species, *A. pictum* consistently had a significantly higher abundance of resident natural enemies, except for August 2019. Our study underlines how perennial flowering plants, such as *A. pictum,* are essential to sustain natural enemy communities and related ecosystem services in arid settings. This work not only informs sustainable pest management initiatives but also shows how non-crop habitats at the periphery of agricultural fields underpin ecological resilience under adverse climatic conditions.

## 1. Introduction

Natural and semi-natural habitats comprise a suite of non-cultivated settings, such as ridges, forests, grasslands, hedgerows, field margins, or fallow land [1]. Natural habitats help to retain invertebrate predators by providing resources, such as shelter, alternative prey, pollen, and nectar, especially in frequently disturbed, ephemeral agroecosystems [2,3,4,5,6]. Predators that thus reside within natural habitats can sustain their populations, colonize nearby (ephemeral) crops, and exert biological control of local pest populations [3,7]. As such, the composition and spatial configuration (or physiognomy) of natural habitats surrounding field crops affect predator activity—abundance patterns [8,9]. By retaining natural habitats within agro-landscape mosaics, predator-mediated biological control is routinely raised, and farm profitability is enhanced [6,10,11]. However, under certain conditions, natural habitat enhances predator populations [12] but does not translate into enhanced levels of pest control [1]. Nevertheless, as the contribution of natural habitat to biological control varies across crop—pest systems, geographies, and time [13], it is essential to carefully assess the underlying ecological determinants and devise habitat management protocols (and crop management regimes) accordingly.

Within natural habitats, plants (in-)directly provide vital (food, non-food) resources and foraging cues for resident predators. Flowering plants directly offer floral nectar but can also provide pollen [14,15,16]. Meanwhile, (non-flowering) plants provide shelter and overwintering habitat [2,4,8] or produce attractant volatiles upon herbivore attacks [17,18]. When present in near proximity to the field, non-crop plants exert important bottom-up effects on predator populations and mediate in-field biological control [2,4,8,19]. By identifying the functional traits of non-crop plants [20] and by accounting for eventual ecosystem disservices (e.g., weediness [21]), ecological engineering tactics can be designed that provide win-win benefits for biodiversity conservation and farmer livelihoods [22,23,24,25]. 

Located in inland Eurasia, China’s Gobi Desert contains a multitude of habitats and harbors a unique insect fauna [26,27,28,29]. In recent years, Gobi oasis habitats have largely been converted to agriculture (to be specific, most for cotton fields and few for orchards, such as pear, jujube, apricot, and *Lycium ruthenicum*). Many agricultural fields lay at the interface between oasis and desert settings and are typified by elevated temperatures, water shortage, sparse vegetation, and fragile ecosystems [27,28,29,30]. Though a diverse set of natural insect enemies can inhabit these types of ecosystems [31,32,33], little is known about the resident biota in China’s Tarim Basin, i.e., an extensive desert in southern Xinjiang that is characterized by extremely sparse vegetation and more severe climatic conditions [26]. The scant knowledge of resident invertebrate predators and their association with local non-crop plants prevents the development of more sustainable forms of pest management. 

In this context, we investigated the floral composition of natural, non-crop habitats at different sites in the Tarim Basin and gauged the identity, relative abundance and temporal dynamics of predatory insects associated with the prevailing plant species. In addition to the above faunal and floral surveys, we explored whether certain plant traits and herbivore abundance are related to either natural enemy identity or relative abundance. By quantitatively assessing the contribution of native plants to biological control in a desert ecosystem, our work provides recommendations on how to design sustainable pest management strategies under climatic uncertainty or variability.

## 2. Materials and Methods

### 2.1. Study Site

**Figure 1:**
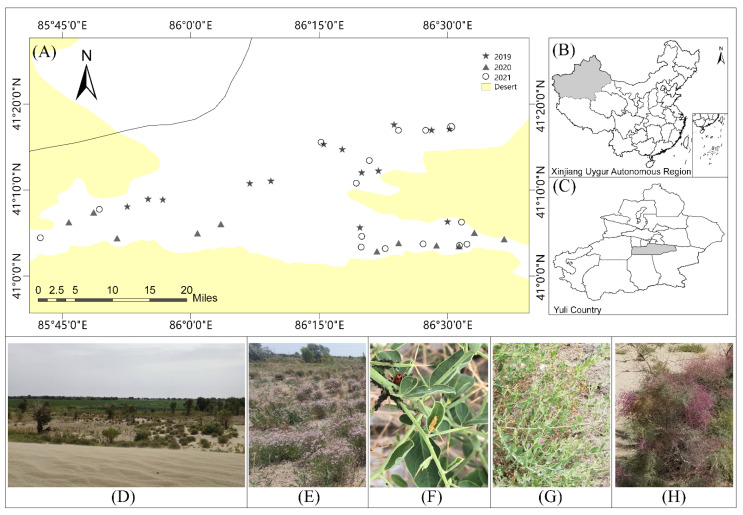
Distribution of field sites within China’s Xinjiang Uygur Autonomous Region from 2019 to 2021, (**A**) distribution of field sites, (**B**) the Xinjiang Uygur Autonomous Region, China, (**C**) Yuli county, (**D**) the Gobi Desert landscape, and some typal vegetations in the Gobi Desert landscape: (**E**) *Apocynum pictum*, (**F**) *Alhagi sparsifolia*, (**G**) *Karelinia caspia*, (**H**) *Tamarix ramosissima*.

Field work was conducted in Yuli County, Xinjiang Uygur Autonomous Region (Figure 1). During 2019–2021, a total of 40 study sites (14, 11, 15 sites for 2019, 2020, 2021, respectively) were chosen 200 m away from arable lands (normally, cotton fields) in the desert-oasis transition zone (i.e., so-called gobi habitat), with more than 1 km between adjacent sites. Sites were representative of the Gobi desert landscape, which is composed of a mosaic of deserts (27.5%–93.5%), oasis settings (semi-natural habitats: 0%–10.9%), and agricultural fields (mainly cotton fields, 5.9%–62.3%), covering 1200 km^2^ in southern Xinjiang (41°03′–41°17′ E, 85°45′–86°36′), and the annual temperature ranged from −22.6 ℃ to 55.2 °C, the relative humidity (RH) was 36.1%, and the average annual precipitation and evaporation were 44 mm and 2700 mm, respectively. All works were conducted within approximately 1 ha at each site to cover most of the vegetation species.

### 2.2. Plant and Predator Survey

At each site, floral surveys were done within five randomly positioned 10 × 10 m quadrats. Within each quadrat, plant species were identified, ground coverage of each species was visually assessed, and phenology (i.e., flowering status: Flowering or not) was recorded by the same observer on a weekly basis from June to August 2019, from May to July 2020, and from May to August 2021. When estimating the proportional coverage of one plant species in the quadrat, the observer estimated the vertical projection area of the upper branches of each cluster, then added them up together to calculate the proportion of the area of this species in the quadrant [34]. The total proportional vegetation coverage of some quadrats may exceed 100% in view of spatial vegetation structure.

At each site, for each plant species, we sampled the arthropods within 5 random plots as 5 replications, which may overlap or not overlap with previous plant quadrats (10 m × 10 m). In each plot, we performed a “five points” type sampling, each point contained a square area of “0.5 m × 0.5 m = 0.25 m^2^”. Sampling was carried out on a weekly basis, from June to August 2019, from May to July 2020, and from May to August 2021. All collected individuals were taken to the laboratory for subsequent taxonomic identification. According to the classification results, the composition of predatory natural enemies on various plants was determined and the abundance of arthropods was calculated as the total number of five points (5 points × 0.25 m^2^ = 1.25 m^2^) for each plot. At last, we calculated the average predator number of five plots (replications) for every plant species at each site and standardized the abundance to the unit area of 1 m^2^ per plot. 

### 2.3. Data Analyses

Given the (two-way) flow of predator populations between arable lands and Gobi habitats, survey data were annually pooled for three periods: May, June to July, and August. Next, to identify the dominant species of foliage-dwelling predators and plants across sites, the Berger–Parker index (*D*) was computed weighting for predator abundance values and relative coverage for plants [35]. The formula is as follows:

Berger–Parker index [35]:

$$D=P_{max}\;$$

in which *P_max_* is the maximum proportion of one species in any sample or the maximum proportion of predator species during any period. Species with *D* ≥ 10% are regarded as dominant species.

Partial redundancy analysis (pRDA) was used to assess the effect of plant traits (i.e., species identity, flowering phenology) and herbivore abundance on the abundance-based community structure of foliage-dwelling predators. We also performed Monte Carlo permutation tests (n = 999) to evaluate the significance of the effects of plant traits and herbivore abundance on the abundance-based predator community structure. The sampling date was entered as a covariant. Predator abundance was transformed by Hellinger’s transformation for negative matches circumventing [36]. 

To emphasize the difference between plant species in the ability to conserve predators, one-way ANOVA and Duncan’s multiple comparison were used to demonstrate the effect of plant identity on the total abundance of predators.

## 3. Results

### 3.1. Floral Composition

Over the entire survey period, the richness of plant species varied from 1 to 6 (3.24 on average), and total vegetation coverage varied from 8% to 140% (54.06% on average) in quadrats. Within the quadrats, a total of 18 plant species were recorded belonging to 18 genera and 10 families (Table 1), with *Apocynum pictum* (Apocynaceae), *Phragmites communis* (Poaceae), *Karelinia caspia* (Asteraceae), and *Tamarix ramosissima* (Tamaricaceae) as the dominant species. Out of all plant species, 83% were perennials.

### 3.2. Predator Composition

**Figure 2:**
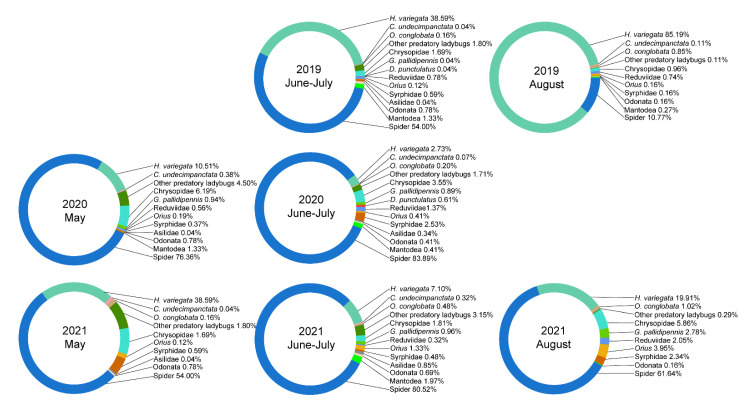
Species composition of invertebrate predators associated with non-crop plants in the Gobi Desert from 2019 to 2021. Each donut chart reveals proportional abundance of one particular species of predator, as determined during three sampling events per year.

During 2019–2021, a total of 10,405 foliage-foraging predators were collected (adult and larvae). These were classified into 9 separate groups and 14 taxa: Ladybeetles (i.e., *Hippodamia variegata*, *Oenopia conglobata*, *Coccinella undecimpunctata*, others), spiders, Chrysopidae, Reduviidae, Anthocoridae (*Orius* spp.), Geocoridae (*Geocoris pallidipennis*), Miridae (*Deraeocoris punctulatus*), Syrphidae, Asilidae, Mantodea, and Odonata. To be mentioned, most of the foliage-dwelling spiders were wandering spider, and the meta-population of spiders contained Philodromidae (i.e., *Philodromus xinjiangensis*), Thomisidae (i.e., *Misumenops tricuspidatus*), Oxyopidae, Gnaphosidae, Lycosidae, Salticidae, and Dictynidae, among others. Important temporal differences were recorded in the species composition of the predator community (Figure 2). Spiders and predatory ladybeetles, especially *H. variegata*, were dominant taxa, with respective relative abundance of 10.8%–83.9%, 4.7%–86.3%, and 2.7%–85.2% (Figure 2). Spiders were the most dominant taxa of the predator community throughout all periods except for August 2019, when the abundance of predatory ladybeetles was much higher than spiders (Figure 2).

### 3.3. Temporal Predator Patterns

**Figure 3:**
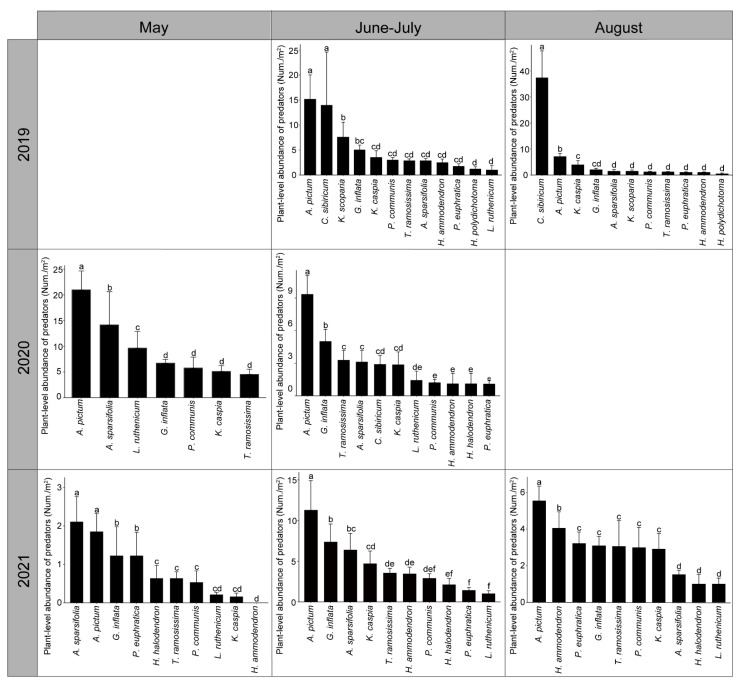
Plant-level abundance of all invertebrate predators. Total abundance levels (mean ± SE) are reported for each plant within 1 m^2^ sampling quadrats. Identical letters above the error bar indicate no statistically significant differences among plant species.

**Figure 4:**
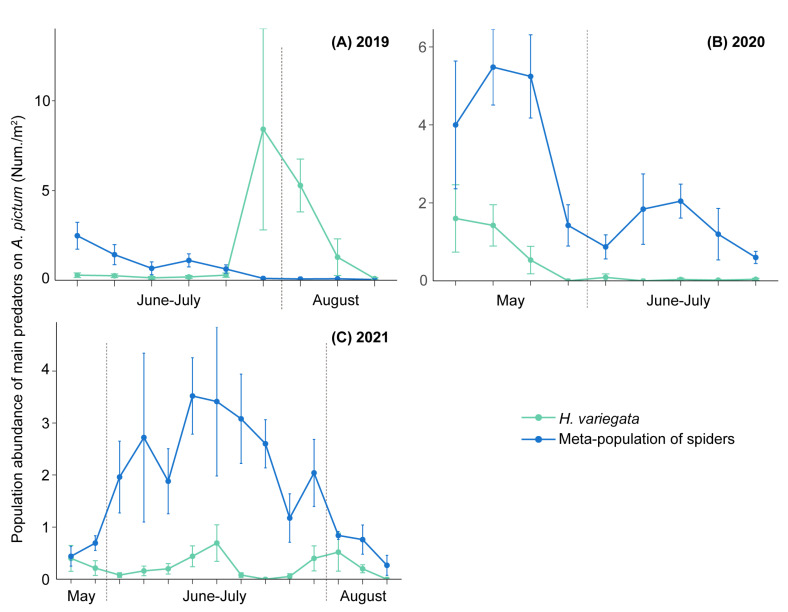
Temporal patterns of the abundance of *H. variegata* and the meta-population of spiders on *A. pictum* from 2019 to 2021. During each sampling event, species-level abundance (mean ± SE) patterns were recorded within 1 m^2^ quadrats.

Over time, plant-level total abundance of invertebrate predators varied to a considerable extent (Figure 3; Table 2). Across sites, *A. pictum* harbored the highest number of predators in all periods except for August 2019 (June–July 2019: *F*_11,78_ = 2.76, *P* = 0.005, May 2020: *F*_6,42_ = 6.86, *P* ˂ 0.001; June–July 2020: *F*_10,53_ = 4.41, *P* ˂ 0.001, May 2021: *F*_9,26_ = 2.66, *P* = 0.025, June–July 2021: *F*_9,56_ = 3.37, *P* = 0.002; August 2021: *F*_9,30_ = 3.42, *P* = 0.005). In August 2019, *C. sibiricum* harbored a higher number of predators than the other plant species (August 2019: *F*_10,66_ = 20.35, *P* ˂ 0.001). In June–July 2019 and May 2021, plant-level predator abundance on *A. pictum* was identical to that on *C. sibiricum* and *A. sparsifolia*, respectively. On *A. pictum*, spiders reached peak abundance levels at an early time, i.e., from May onward in 2020 and from late June to early July in 2021. However, *H. variegata* populations were marked by three distinct peaks in 2021, i.e., during May, from late June to early July, and from late July to mid-August, and by only one peak in other years, i.e., from late July to mid-August 2020 and May 2020 (Figure 4).

### 3.4. Plant Effects on Resident Predator Community

Plant variables had significant effects on the predator community (Table 3), with species identity, herbivore abundance, and flowering status explaining 3%–44% of the total variance of the predator community (R_adj_). Monte Carlo permutation tests showed that plant species identity determined the resident predator community during all sampling periods (June–July 2019: *F*_13,408_ = 8.12, *P* ˂ 0.001, August 2019: *F*_13,193_ = 4.10, *P* ˂ 0.001, May 2020: *F*_10,128_ = 2.76, *P* ˂ 0.001, June–July 2020: *F*_10,300_ = 1.96, *P* ˂ 0.001, May 2021: *F*_9,49_ = 2.83, *P* ˂ 0.001, June–July 2021: *F*_9,307_ = 23.88, *P* ˂ 0.001, August 2021: *F*_19,125_ = 4.10, *P* ˂ 0.001). The herbivore community was mainly composed of Aphididae, Psyllidae, Curculionidae, Chrysomeloidea, phytophagous mirid bugs, Thysanoptera, and some larvae of Lepidoptera, and their abundance exhibited a similar trend in 2020 and 2021 (May 2020: *F*_1,137_ = 29.11, *P* ˂ 0.001, June–July 2020: *F*_1,205_ = 63.10, *P* ˂ 0.001, May 2021: *F*_1,57_ = 7.76, *P* = 0.005, June–July 2021: *F*_1,310_ = 7.43, *P* < 0.001, August 2021: *F*_1,133_ = 7.43, *P* ˂ 0.001). Lastly, flowering status only affected the predator community in May 2021 (*F*_1,57_ = 3.39, *P* = 0.023) and August 2021 (*F*_1,133_ = 4.43, *P* ˂ 0.015). 

**Figure 5:**
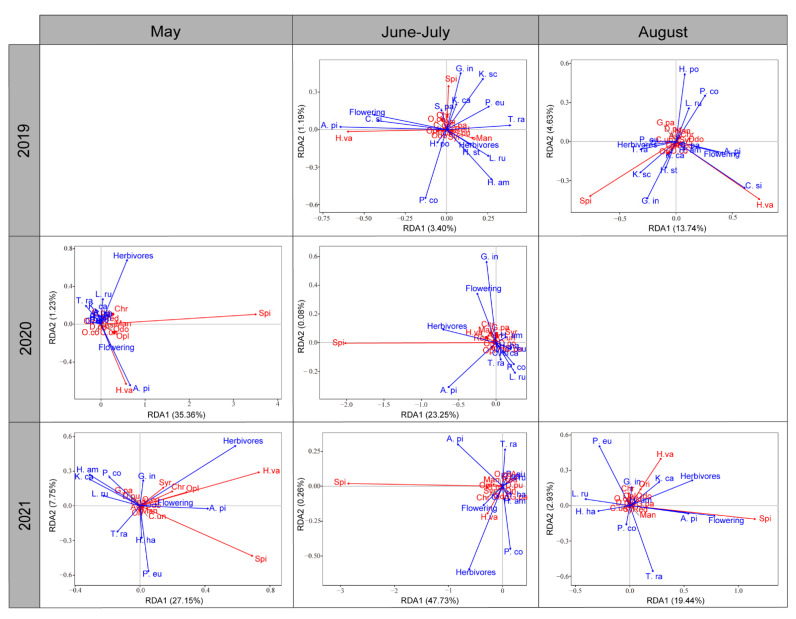
Partial redundancy analysis showing the extent to which plant traits and herbivore abundance affect predator abundance and diversity. In each biplot, explanatory variables are shown in blue characters and arrows, while response variables are displayed in red. Each plant and arthropod species are indicated with the first three letters of its scientific name. “Opl” indicates “other predatory ladybeetles”. Plant phenology “phe” captures the flowering status i.e., “phe1”-flowering or “phe0” not flowering, while “pest” refers to total pest abundance. *A. sparsifolia* is used as a reference for all other plant species.

According to biplots (Figure 5), the abundance of *H. variegata* was higher on *A. pictum* (2019–2021), *C. sibiricum* (2019), and *P. euphratica* (August 2021), and it was positively correlated with flowering status (2019–2021) and herbivore abundance (2020–2021). Spider abundance was higher on *G. inflata* and *K. caspia* (2019), *A. pictum* (2020), *H. halodendron*, *K. caspia*, and *P. euphratica* (May 2021) and *T. ramosissima* (August 2021), and it was positively correlated with flowering status and herbivore abundance in 2020–2021.

## 4. Discussion

Biological control, i.e., the scientifically-guided conservation, augmentation, or release of beneficial organisms within agricultural settings, constitutes a sound, practicable, and cost-effective approach to managing crop pests, weeds, and pathogens [37,38,39,40]. By consciously implementing biological control in the world’s farming systems, pest-related crop losses can be minimized, and a myriad of societal benefits can be gained [2,37,38,39]. Invertebrate predators are prime providers of biological control services and thus a prominent feature for more sustainable forms of pest management [41,42,43]. By conserving or (periodically) enhancing invertebrate predators in agricultural landscapes, pest outbreaks can be averted [41,44,45,46] and pesticide use can be curbed [22,47,48]. Although mono-cropping, chemical intensification, and the systematic removal of non-crop habitats have enhanced crop yields in many farming systems, they have also negatively impacted resident predator communities and degraded their associated biological control services [49,50]. To counter these processes and mitigate their negative societal impacts [51], ecologically based pest management strategies are increasingly adopted, and schemes are deployed to bolster predator-mediated biological control within farming landscapes [3,52].

Non-crop plants (and habitats) in the vicinity of agricultural fields can play an important role in sustainable pest management by acting as a source of natural enemy populations and by providing vital food and non-food resources [2,20]. In oasis habitats in China’s Gobi Desert, seven taxa of agriculturally important predators are commonly found in non-crop settings: *H. variegata*, *O. conglobata*, *C. undecimpunctata*, Chrysopidae, *Orius* spp., *G. pallidipennis*, and *D. punctulatus*. Similar observations have been made in northern Xinjiang [47,48,49], and other studies have shown how species, such as *Philodromus xinjiangensis* (Araneae; Philodromidae), *Chrysopa sinica*, *C. phyllochroma*, *C. pallens* (Chrysopidae), and *Syrphus corolla* (Syrphidae), inhabit these habitats [32,52]. Ladybeetles, especially *H. variegata*, and spiders, which are mainly composed of wandering spiders, such as Philodromidae (i.e., *Philodromus xinjiangensis*), Thomisidae (i.e., *Misumenops tricuspidatus*), Oxyopidae, Gnaphosidae, Lycosidae, Salticidae, and Dictynidae, are hereby the dominant organisms in farmland and non-crop settings.

Ladybeetles are key predators in farming systems across the globe, where they prey upon aphids, plant lice, mites, and other soft-bodied insects [53,54,55]. However, they also engage in intraguild predation and cannibalism to a varying extent [56]. Similarly, spiders prey on a broad suite of crop pests and can rapidly colonize a newly-established crop (e.g., through ballooning [57]) but also feed on other natural enemies [58]. While a higher diversity and abundance of the above predators does not necessarily translate into enhanced biological control [1,58,59], the presence of flowers and overall habitat diversification can possibly attenuate some of the negative side-effects of agricultural intensification [60]. Irrespective of the potential trade-offs (see also [21]), the protection, restoration, and establishment of natural habitat patches interspersed among crop fields will benefit resident natural enemy populations and potentially lower pest-induced crop losses.

According to the ‘resource concentration hypothesis’, landscape-level diversification (e.g., through a crop—non-crop habitat mosaic) can lower pest densities [61]. Meanwhile, non-crop habitats of adequate structure, architectural complexity, and plant species composition can provide critical resources to resident natural enemies. Much can be gained in terms of biological control by pairing temporally stable non-crop and ephemeral crop habitats [62]. Within natural habitats, non-crop plant species contribute to biological control to varying extent, and different traits (e.g., floral and extra-floral nectaries) mediate the plant’s value to foraging natural enemies [63,64]. The relative importance of these traits is further mediated by the identity and development stage of resident natural enemies. Occasionally, one single plant species can dictate natural enemy community composition and the resulting biological control outcomes [65]. In the Gobi Desert oases, predatory ladybeetles and spiders are omnivores that may benefit greatly from access to non-crop plants. Predatory ladybeetles can utilize nectar and pollen and can thus increment their life span and fertility by accessing flower resources [64]. Similarly, while spiders are largely thought to be exclusively carnivorous, they gain fitness benefits from feeding upon extra-floral nectar, and some cursorial spiders are even attracted to nectar odors [64,66]. Natural enemies, such as hymenopteran parasitoids, also gain major fitness benefits from access to (floral, extra-floral) nectar and pollen (e.g., [67]), and future work can investigate the extent to which Gobi Desert plants add to parasitoid-mediated biological control.

In our study, plant species identity, flowering status, and herbivore abundance all determined the natural enemy community, but the explanatory power was low. Overall, the explanatory power was highest during early- and mid-season but consistently low in late-season. Given the marked early-season population peak of ladybeetles on *A. pictum* (Figure 4), this plant species can provide a clear advantage for biological control and facilitate their ensuing crop colonization [3,68,69]. On the other hand, during late season, plant-level predator abundance in natural habitats is likely shaped by predator spillover from nearby crops. More specifically, the populations of predatory ladybeetles are donor-controlled and highly responsive to increasing pest numbers, e.g., in neighboring cotton crops. For large-bodied natural enemies, such as ladybeetles and spiders, high immigration rates during early season are of critical importance in ensuring pest suppression [70]. Hence, non-crop plants that favor these predators during early- and mid-season (e.g., *A. pictum*, *C. sibiricum*, and *A. sparsifolia*) can play a pivotal role in biological control in Gobi Desert settings. Other traits, such as plant height, can further facilitate early-season predator colonization or spider ‘mass action’ [71]. Therefore, the presence of tall non-crop plants, such as *T. ramosissima*, in the landscape matrix could be hereby essential.

A valuable trait of functional plants is the flower structure in which open-nectar flowers provide readily accessible carbohydrates for foraging predators [72,73]. This is somewhat corroborated by our findings, as plants like *A. pictum* or *C. sibiricum* have open-nectar flowers and support large numbers of predators. Meanwhile, plants with concealed nectar, like *A. sparsifolia,* can also play a role under suitable conditions, but their relative role is mediated by natural enemy size and other traits, e.g., proboscis length [20]. While the latter plants may offer limited nutritional benefits to spiders or ladybeetles, their nectar resources could be exploited by smaller organisms, such as mirids.

The broader usage value, ecosystem disservices, and relative (maintenance) costs of non-crop plants can dictate farm-level adoption and diffusion of habitat management schemes. Among the 19 desert plant taxa surveyed in our study, *A. pictum* not only conserves resident predators but is also locally valued for tea production and traditional medicine [74,75]. This differs from other plants, such as *C. sibiricum* and *A. sparsifolia*, which provide disservices and are well-recognized farmland weeds. While flowering annuals can support natural enemies and contribute to biological control in adjacent cropland [68], perennials may be of considerable interest from a (pest) management perspective. In addition to their proven benefits for multiple natural enemies [68,76,77], temporally continuous habitats with perennials require less maintenance (and establishment costs) as compared to, for example, strips with annual flowering plants [63]. The former may even outperform annuals in natural enemy conservation [63]. As these advantages possibly are more pronounced under adverse climatic conditions (e.g., desert settings), the use of perennials in habitat management schemes could be considered to sustain biological pest control under climate change scenarios.

## 5. Conclusions

In this study, we investigated the floral composition of the Gobi Desert at different sites in the Tarim Basin and gauged the identity, relative abundance, and temporal dynamics of predatory insects associated with the prevailing plant species. We also explored whether certain plant traits and herbivore abundance are related to either natural enemy identity or relative abundance. Our results demonstrate that *Apocynum pictum* (Apocynaceae), *Phragmites communis* (Poaceae), *Karelinia caspia* (Asteraceae), and *Tamarix ramosissima* (Tamaricaceae) are the dominant species of the vegetation community of the Gobi Desert, predatory ladybeetles and spiders are the dominant predators that are routinely associated with the vegetation of the Gobi Desert, and plant traits (i.e., plant identity and flowering status) and herbivore abundance explained up to 44% (3%–44%) of the variation in natural enemy community and exhibited consistent, year-round effects. Among all plant species, *A. pictum* consistently had a higher abundance of resident natural enemies except for August 2019. Our study underlines how perennial flowering plants, such as *A. pictum,* are essential to sustain natural enemy communities and related ecosystem services in arid settings. By quantitatively assessing the contribution of native plants to biological control in a desert ecosystem, our work provides recommendations on how to design sustainable pest management strategies under climatic uncertainty or variability.

## Figures and Tables

**Table 1:** Plant species trait and relative coverage.

Family	Species	Flowering Phase	Life	Flower	Coverage Proportion			Dominance
			Cycle	Structure	2019	2020	2021	Level
Apocynaceae	*Apocynum pictum*	Early-May to unknown time	P	O	11.33%	9.91%	6.06%	Ds
Asclepiadaceae	*Cynanchum sibiricum*	Mid-May to unknown time	P	O	3.01%	6.56%	0.23%	
Asteraceae	*Hexinia polydichotoma*	Mid-May to early-August	P	O	0.73%	<0.01%	<0.01%	
	*Inula salsoloides*	Late-June to early-August	P	O	0.15%	<0.01%	—	
	*Karelinia caspia*	Late-June to unknown time	P	O	9.98%	10.36%	19.01%	Ds
	*Scorzonera divaricata*	Late-June to late-July	P	O	<0.01%	<0.01%	—	
Chenopodiaceae	*Haloxylon strobilaceum*	Early-August to unknown time	P	O	0.08%	<0.01%	3.30%	
	*Halocnemum ammodendron*	Early-August to unknown time	P	O	3.70%	0.21%	6.50%	
	*Kochia prostrata*	Late-July to unknown time	P	O	1.66%	—	—	
	*Suaeda paradoxa*	Late-August to unknown time	P	O	0.15%	—	—	
Elaeagnaceae	*Elaeagnus angustifolia*	Mid-May to early-June	P	O	—	0.29%	—	
Gramineae	*Phragmites communis*	Late-August to unknown time	A	O	28.27%	46.23%	11.76%	Ds
Leguminosae	*Alhagi sparsifolia*	Mid-May to mid-July	P	C	8.19%	3.84%	4.12%	
	*Glycyrrhiza inflata*	Mid-June to late-July	P	C	5.47%	4.54%	9.02%	
	*Halimodendron halodendron*	Mid-May to early-June	P	C	<0.01%	0.25%	1.17%	
Tamaricaceae	*Tamarix ramosissima*	Mid-May to unknown time	P	O	19.10%	11.81%	20.38%	Ds
Salicaceae	*Populus euphratica*	Unrecorded	P	O	6.51%	3.72%	9.70%	
Solanaceae	*Lycium ruthenicum*	Mid-June to mid-July	P	O	1.65%	2.27%	8.74%	

Note: “—” demonstrate this vegetation taxon was not sampled during a given year. In the description of life cycle and flower structure of the plant species, “A” and “P” stand for annual and perennial, respectively, in addition “C” and “O” represent “concealed-nectar” or “open-nectar”. “Ds” indicates that this plant species is a dominant taxon in the vegetation community of the Gobi Desert.

**Table 2:** One-way ANOVA on the effects of plant species on total abundance of foliage-dwelling predators.

Year	Period	Factors	*df_1_*	*df_2_*	*F*	*P*
2019	June–July	Plant species	11	78	2.76	0.005
	August		10	66	20.35	˂0.001
2020	May	Plant species	6	42	6.86	˂0.001
	June–July		10	53	4.41	˂0.001
2021	May	Plant species	9	26	2.66	0.025
	June–July		9	56	3.37	0.002
	August		9	30	3.42	0.005

**Table 3:** Effects of plant traits and herbivore abundance on the resident insect predator community.

Year	Period	Factors	*Df_1_*	*Df_2_*	*F*	*P*	R_adj_
2019	June–July	Plant species	13	408	2.10	˂0.001	0.03
		Herbivore abundance	1	421	0.85	0.469	
		Flowering status	1	421	0.36	0.865	
	August	Plant species	13	193	3.33	˂0.001	0.11
		Herbivore abundance	1	206	0.84	0.511	
		Flowering status	1	206	0.15	0.978	
2020	May	Plant species	10	128	4.40	˂0.001	0.29
		Herbivore abundance	1	137	29.11	˂0.001	
		Flowering status	1	137	0.28	0.756	
	June–July	Plant species	10	300	5.38	˂0.001	0.20
		Herbivore abundance	1	205	63.10	˂0.001	
		Flowering status	1	205	1.53	0.192	
2021	May	Plant species	9	49	2.83	˂0.001	0.31
		Herbivore abundance	1	57	7.76	0.005	
		Flowering status	1	57	3.39	0.023	
	June–July	Plant species	10	307	23.88	˂0.001	0.44
		Herbivore abundance	1	310	38.70	˂0.001	
		Flowering status	1	310	2.75	0.074	
	August	Plant species	9	125	3.52	˂0.001	0.19
		Herbivore abundance	1	133	7.43	˂0.001	
		Flowering status	1	133	4.43	0.015	

## Data Availability

All data analyzed in this study are included in this article.
